# Toll-like receptor 3 in Epstein-Barr virus-associated nasopharyngeal carcinomas: consistent expression and cytotoxic effects of its synthetic ligand poly(A:U) combined to a Smac-mimetic

**DOI:** 10.1186/1750-9378-7-36

**Published:** 2012-12-03

**Authors:** Benjamin Vérillaud, Mélanie Gressette, Yannis Morel, Carine Paturel, Philippe Herman, Kwok Wai Lo, Sai Wah Tsao, Michel Wassef, Anne-Sophie Jimenez-Pailhes, Pierre Busson

**Affiliations:** 1 CNRS-UMR 8126, Institut de Cancérologie Gustave Roussy, University Paris-Sud 11, 39 rue Camille Desmoulins, 94805, Villejuif cedex, France; 2 Department of Head and Neck surgery, Lariboisière Hospital, AP-HP, University Paris-Diderot Paris 7, Paris, France; 3 Innate Pharma, Marseille, France; 4 Department of Anatomical and Cellular Pathology, State Key Laboratory in Oncology in South China, Prince of Wales Hospital, The Chinese University of Hong Kong, Ma Liu Shui, Hong Kong; 5 Department of Anatomy, the University of Hong Kong, Pok Fu Lam, Hong Kong; 6 Department of Pathology, Lariboisière Hospital, AP-HP, University Paris-Diderot Paris 7, Paris, France

**Keywords:** Toll-Like Receptor 3, Nasopharyngeal cancer, Epstein-Barr virus, Poly(A:U), Smac-mimetic, Inhibitor of Apoptosis Protein

## Abstract

**Background:**

Nasopharyngeal carcinomas (NPC) are consistently associated with the Epstein-Barr virus (EBV). Though NPCs are more radiosensitive and chemosensitive than other tumors of the upper aero-digestive tract, many therapeutic challenges remain. In a previous report, we have presented data supporting a possible therapeutic strategy based on artificial TLR3 stimulation combined to the inhibition of the IAP protein family (Inhibitor of Apoptosis Proteins). The present study was designed to progress towards practical applications of this strategy pursuing 2 main objectives: 1) to formally demonstrate expression of the TLR3 protein by malignant NPC cells; 2) to investigate the effect of poly(A:U) as a novel TLR3-agonist more specific than poly(I:C) which was used in our previous study.

**Methods:**

TLR3 expression was investigated in a series of NPC cell lines and clinical specimens by Western blot analysis and immunohistochemistry, respectively. The effects on NPC cells growth of the TLR3 ligand poly(A:U) used either alone or in combination with RMT5265, an IAP inhibitor based on Smac-mimicry, were assessed using MTT assays and clonogenic assays.

**Results:**

TLR3 was detected at a high level in all NPC cell lines and clinical specimens. Low concentrations of poly(A:U) were applied to several types of NPC cells including cells from the C17 xenograft which for the first time have been adapted to permanent propagation *in vitro*. As a single agent, poly(A:U) had no significant effects on cell growth and cell survival. In contrast, dramatic effects were obtained when it was combined with the IAP inhibitor RMT5265. These effects were obtained using concentrations as low as 0.5 μg/ml (poly(A:U)) and 50 nM (RMT5265).

**Conclusion:**

These data confirm that TLR3 expression is a factor of vulnerability for NPC cells. They suggest that in some specific pathological and pharmacological contexts, it might be worth to use Smac-mimetics at very low doses, allowing a better management of secondary effects. In light of our observations, combined use of both types of compounds should be considered for treatment of nasopharyngeal carcinomas.

## Background

The TLRs are type-I transmembrane proteins which play a key role in the detection of pathogens and in triggering inflammation and immune response to microbial infections [1]. The stimulation of TLRs by their respective ligands initiates well-characterized signaling cascades that enhance cellular resistance against pathogens. TLRs are expressed not only by immune cells, but also by various cell types including normal and malignant epithelial cells [2,3]. TLR3 is one category of TLR which interact specifically with double-stranded RNA (dsRNA) of viral origin. This binding leads in turn to MAPKs, NF-kB and IRF-3 activation and to IFN-I induction via the adaptor proteins TRIF and RIPK1 [1,4-6]. Activation of these pathways results in the establishment of an anti-viral phenotype. Many additional changes are induced by TLR3 stimulation. Their overall consequences are dependent on the intensity of the stimulation, on the cell context and on concomitant extra-cellular signals. Depending on these conditions, they can result in apoptotic cell death as well as enhancement of proliferation [7-10].

Nasopharyngeal carcinoma (NPC) is a malignant tumor arising from the epithelial lining of the nasopharynx. Consistently associated with the Epstein-Barr virus (EBV), NPC is the third most frequent virus-associated malignancy in humans [11,12]. Foci of high incidence (25 cases per 100,000 individuals per year) are localized in South-East Asia, especially in the Guangdong and Guangxi provinces of Southern China. There are also large areas of intermediate incidence (3 to 8 cases) in South-East Asia (Indonesia) and Northern Africa (Tunisia, Algeria and Morocco) [12]. The multifactorial pathogenesis of NPC relies on germ-line genetic susceptibility, acquired cellular genetic and epigenetic alterations - especially under the influence of diet carcinogens - and EBV infection [12-14]. Although most viral genes are silent in NPC cells, some are consistently expressed including those encoding the nuclear protein EBNA1 (Epstein-Barr Nuclear Antigen 1), two membrane proteins named LMP1 and LMP2 (Latent Membrane Proteins 1 and 2) and the short untranslated RNAs EBER1 and EBER2. Of the very large amounts of EBERs produced and present in the nuclei of NPC cells, some are exported in the cytoplasm and possibly in the extra-cellular space [15]. Local inflammation is also suspected to play a major role in NPC cell growth because of the consistent presence of a massive lymphoid infiltrate in the primary tumor and the intense local production of inflammatory cytokines [16,17]. Surprisingly, to our knowledge, there has been no report so far about the expression of TLR3 in EBV-related nasopharyngeal carcinomas.

In a previous publication, we have reported that malignant NPC cells are highly sensitive to a treatment combining a TLR3 artificial ligand (poly(I:C)) and a Smac-mimetic, RMT5265 which inhibits several proteins of the IAP family (Inhibition of Apoptosis Proteins; namely c-IAP1, c-IAP2 and XIAP) [18]. However poly(I:C) is not strictly specific of TLR3; it interacts with other cellular receptors of double-strand RNAs, especially RIG-I and MDA5 [19]. This lack of specificity might result in undesirable side-effects when used in humans. Therefore, we undertook to investigate the anti-tumoral effects of poly(A:U) in the same experimental context. Poly(A:U) is a TLR3 agonist which does not interact with RIG-I and MDA5 and which is believed to be better tolerated in medical use [19].

The aims of the present study were to confirm the expression of the TLR3 protein in NPC cells and to assess their sensitivity to poly(A:U) as a single agent and in combination with RMT5265. We report that TLR3 is consistently detected in all investigated NPC cell lines and specimens. We also show that various NPC cell lines are exquisitely sensitive to this combined treatment in both high density cultures and clonogenic assays.

## Materials and methods

### Xenografts and cell lines

The following NPC cell lines were used throughout the study: C666-1, C15, C17, C18 (EBV positive cell lines), CNE1 and HONE1 (EBV negative cell lines). A panel of non-NPC epithelial malignant and non malignant cell lines was used as control for TLR3 expression assessment: FaDu and SQ20B (head and neck squamous cell carcinoma, HNSCC), HeLa (cervical carcinoma), A431 (vulvar squamous carcinoma), A549 (non-small cell lung cancer), C33 and CaSki (cervical carcinomas), NP69 and NP460 (derived from non-malignant nasopharyngeal epithelial cells immortalized by SV-40 infection and stable transfection of the gene encoding the telomerase catalytic subunit hTert, respectively) [20-26]. The non-malignant nasopharyngeal NP69 cell line was also used as a control for proliferation assays.

C666-1 cells are EBV-positive NPC cells which have been grown for a long time either as xenografted tumors or *in vitro* cultures [27]. Through this study, we used C666-1 cells stably transfected with the luciferase 1 gene which were kindly provided by Dr Fei-Fei Liu (university of Toronto, Ontario, Canada) [27,28]. These cells retain the EBV genome and intense expression of the EBER viral non-coding RNAs (see the result section). Because the luciferase gene is very stable in these cells both *in vitro* and *in vivo*, it allows *in vivo* imaging of the xenografted tumors. Therefore, we chose to use them from the beginning in anticipation of future *in vivo* studies about the effects of TLR3 agonists on NPC cells. C666-1 cells were routinely propagated *in vitro* using RPMI 1640 medium (Gibco-Invitrogen, Carlsbad, CA) supplemented with 25 mM HEPES and 7.5% fetal calf serum (FCS), in plastic flasks coated with collagen I (Biocoat; Becton-Dickinson, Franklin Lakes, NJ). C15, C17 and C18 are EBV-positive NPC xenografts propagated by subcutaneous passages into nude mice [29]. For a long time, it has not been possible to derive long-term *in vitro* cultures from any of these three xenografts. However, we recently adapted C17 cells to permanent *in vitro* propagation using a protocol inspired from Liu et al. [30]. Briefly, C17 xenografted tumors were minced and treated with type II collagenase for cell dispersion as previously reported [16]. Cells were then plated on a non-irradiated feeder layer of Normal Human Dermal Fibroblasts (NHDF; Promocell, Heidelberg, Germany) and grown in RPMI 1640 medium (Gibco-Invitrogen) supplemented with 25 mM HEPES, 7.5% fetal calf serum (FCS), and 7 μmol/L of the Rho kinases I and II inhibitor Y-27632 (Y-27632; Enzo Life Sciences, Lausen, Switzerland) [31]. Feeder cells became rapidly senescent. Most of them were already eliminated beyond the third *in vitro* passage. For cytological analysis, C17 cells were stained with hematoxilin and eosin safran (HES) after cytospin preparation. Detection of the EBERs by in situ hybridization on C666-1, HeLa, and C17 cell pellets was performed using the INFORM EBER Probe (Ref 800–2842) and the ISH iVIEW Blue Detection Kit (Ref 800–092) from Ventana-Roche (Tucson, AZ). EBV-negative cell lines CNE1 and HONE1 were grown in RPMI 1640 medium (Gibco-Invitrogen) supplemented with 5% FCS [32,33]. NP69 cells were grown in keratinocyte serum-free medium (Gibco) supplemented with 10% FCS.

### Clinical specimens and immunohistochemistry

Biopsies were obtained from 10 patients referred to the Lariboisière hospital (Paris, France). All patients had non-keratinizing undifferentiated (or type III) NPC according to the WHO classification (2005). Biopsies were fixed in formaldehyde and paraffin-embedded. Tissue sections were microwaved at 98°C for 30 minutes in citrate buffer (10 mM, pH 7.3) and then incubated with an antihuman TLR3 mouse monoclonal antibody (40 F9.6, Innate Pharma, Marseille). Binding of the primary antibody was detected with the CSA II kit from Dako (based on a tyramide amplification system; DakoCytomation, Glostrup, Denmark). C666-1 and NP69 cell pellets embedded in paraffin were used for positive and negative control of TLR3 immunostaining. All the clinical samples were obtained and processed according to the guidelines of Lariboisière hospital institutional review board. requiring written informed consent from patients for publication.

### Treatments of cells with pharmacological reagents

The polycyclic C2-symmetric (40 carbon atoms) compound RMT5265 mimics the three-dimensional structure of the N-terminal tetrapetide of Smac/Diablo (second mitochondria–derived activator of caspases) [34]. This compound was kindly provided by Xiaodong Wang, Dallas. It was dissolved in DMSO. The TLR3 agonists - poly(I:C) and poly(A:U) - were obtained from InvivoGen (San Diego, CA). Cisplatinum was purchased from Sigma Aldrich (St. Quentin Fallavier, France).

### Cell growth and viability assays

Cell viability was determined in a short-term assay based on the reduction of MTT (CNE1, HONE1, NP69, C17) or WST (a soluble form of MTT; C666-1). MTT and WST were purchased from Sigma Aldrich. For this assay, cells were seeded in 96-well plates at a density of 2 x 10^3^ (CNE1, HONE1, NP69) or 3 × 10^4^ (C666-1, C17) cells per well. The MTT/WST reaction was performed after 72 hours of culture. The absorbance (Optical Density (OD)) was measured at 550 nm and 450 nm for MTT and WST assays, respectively. The percentage of inhibition was determined based on the difference of OD between treated and untreated cells, after subtraction of the optical background.

Alternatively, *in vitro* growth assays at low density were performed to evaluate the clonogenic potential of NPC cells. HONE1, CNE1 and NP69 cells were plated in six-well plates (5x10^2^ cells per well) and treated at day 1 with RMT5265 and/or poly(I:C) / poly(A:U). After 2 weeks of culture, cell colonies were stained with a solution of Crystal Violet (Sigma Aldrich) in methanol. The clonal growth of C666-1 NPC cells was assessed using a feeder layer of Normal Human Dermal Fibroblasts (NHDF, Promocell). The first day of clonogenic assay, NHDF were plated in six-well plates (1.5x10^5^ per well). 24 hours later, they were irradiated (40 Gy) and C666-1 NPC cells were added at a density of 5x10^3^ cells per well. After 24 hours, once the epithelial cells had firmly adhered to the plate, RMT5265 and/or poly(I:C) / poly(A:U) were added to the culture medium. Replacement by fresh medium was done once a week. After 2 to 4 weeks of culture, cell colonies were stained with a solution of Rhodanile Blue (Sigma Aldrich) in ethanol. Dried plates were then scanned and digitized to allow optical magnification and precise counting of cell colonies.

We used the Bliss additivism model to quantify the level of synergy in our drug-combination experiments [35]. The predictive Bliss additive effect *C* of two single compounds with effects *A* and *B* is: *C* = *A* + *B* - *A*·*B*. The excess over Bliss additivism (EOBA) was calculated by subtracting the predicted Bliss additive effect from the experimentally observed inhibition. It is expressed as a percentage, and represents the excess of inhibition over the predicted response that was obtained when 2 compounds were used in combination.

### Assessment of PARP cleavage

Apoptosis was evaluated by the detection of the cleavage of poly(ADP-ribose)polymerase (PARP) by Western blot analysis performed on total cell protein extracts (see next paragraph).

### Cell protein extraction and western blot analysis

Proteins from cultured cells or xenografts were extracted by lysis in RIPA buffer (50 mM Tris, 150 mM NaCl, 5 mM EDTA, 0.5% sodium deoxycholic acid, 0.5% NP-40, 0.1% SDS) supplemented with a protease inhibitor cocktail (Complete; Roche Molecular, Neuilly sur Seine, France). They were separated by SDS-PAGE and transferred to polyvinylidene difluoride membranes (Immobilon, Millipore, Billerica, CA) by electroblot at 4°C for 90 minutes at 90 V or overnight at 45 V. The antibodies used for Western blot analysis were mouse monoclonal antibodies directed against the human TLR3 (clone 512505, ref. MAB1487, R&D Systems), PARP (C-2-10, ref. 53643; Santa Cruz Biotechnology), β-Actin (AC-74; Sigma Aldrich) and α-Tubulin (B-5-1-2; Sigma Aldrich). Blotted membranes were incubated with a secondary peroxidase-conjugated antibody, and chemiluminescent detection was done using the Immobilon Western Chemiluminescent HRP Substrate (Millipore, Billerica, CA). Specific protein bands were quantified using ImageQuant TL software (GE Healthcare Bio-Sciences AB, Uppsala, Sweden) when the acquisition was performed with ImageQuant LAS 4000 mini biomolecular imager (GE Healthcare Bio-Sciences AB), and a GS-710 calibrated imaging densitometer with Quantity One software (Biorad, Marnes la Coquette, France) when detection was performed on chemiluminescence films.

## Results

### TLR3 is constitutively expressed by NPC cells

**Figure 1:**
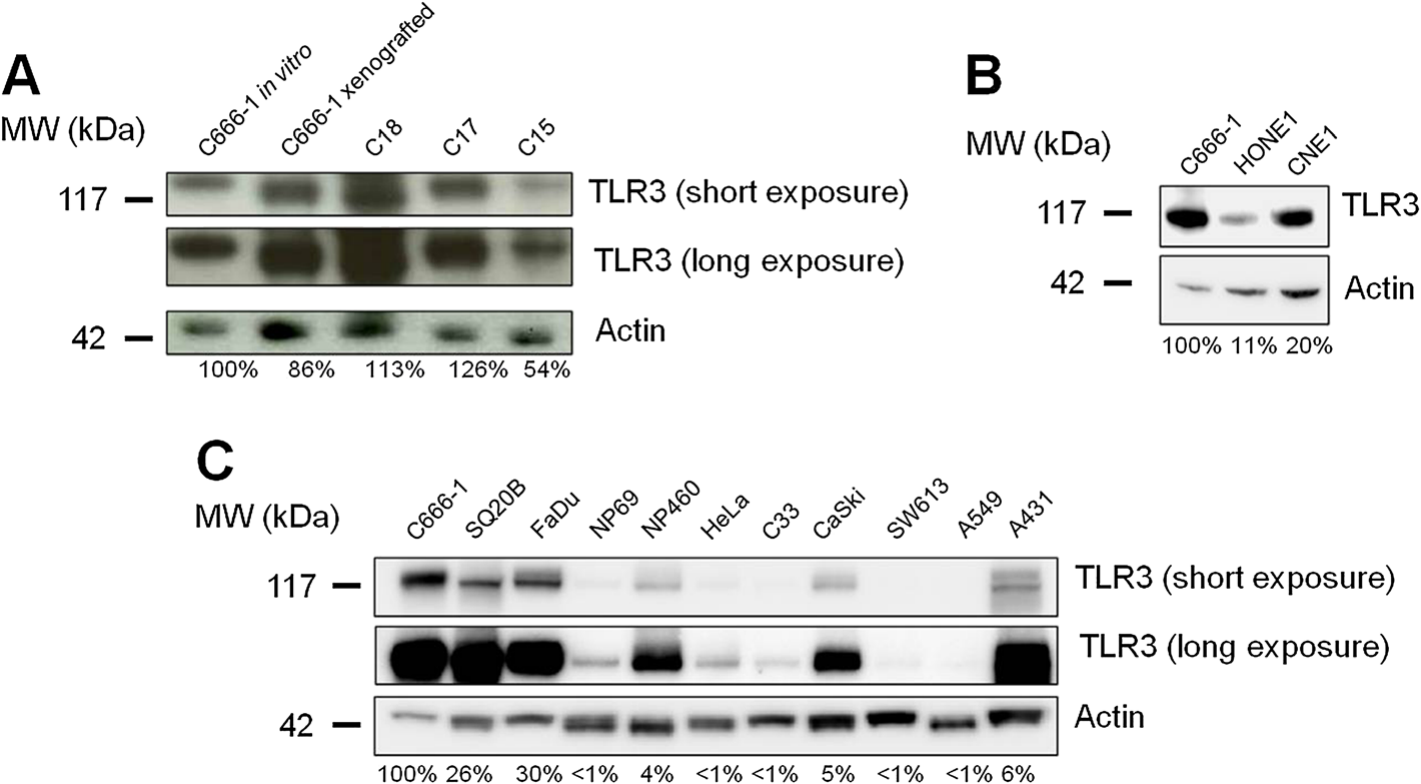
**TLR3 is highly expressed in NPC cell lines and xenografts.** Western blot analysis reveals intense expression of TLR3 in EBV-positive C666-1, C18, C17 and C15 xenografts/cell lines (**A**), and in EBV-negative HONE1 and CNE1 NPC cell lines (**B**). To better characterize the status of TLR3 in NPC cells, its expression was simultaneously assessed in C666-1, in the non NPC head and neck squamous cell carcinoma cell lines SQ20B and FaDu, and in a panel of non malignant and malignant epithelial cell-lines, namely NP69 and NP460 (non tumorigenic immortalized nasopharyngeal cell lines), HeLa, C33 and CaSki (cervical carcinoma), SW613 (colon carcinoma), A549 (non-small cell lung cancer), and A431 (vulvar squamous carcinoma) (**C**). The same blotted membranes were stained with anti-actin for protein loading controls.

**Figure 2:**
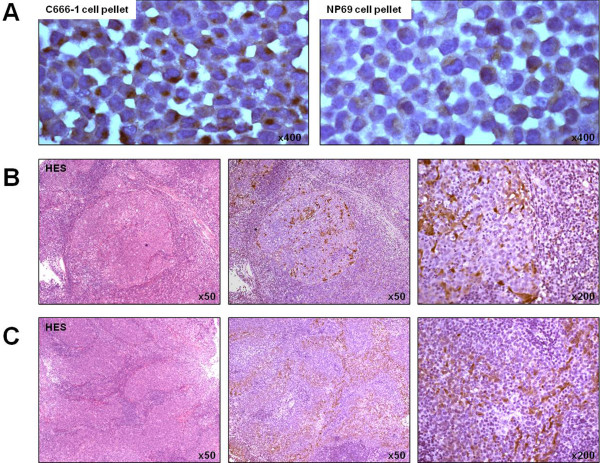
**TLR3 is consistently detected by immunohistochemistry on tissue sections from NPC biopsies.** (**A**) C666-1 and NP69 cell pellets were used as positive and negative controls for TLR3 immunostaining, respectively; note the strong juxtanuclear staining in C666-1 cells (suggestive of endosomal TLR3 localization) and the low level of staining in NP69 cells, consistent with western blot data displayed in figure 1C. (**B**,**C**) NPC biopsy sections (B = patient#6; C = patient#8) were stained with hematoxilin and eosin safran (HES), or immunostained with antihuman TLR3 antibody (40 F9.6, Innate Pharma, Marseille). TLR3 was detected in all biopsies in a substantial fraction of cells, although with heterogeneous levels of intensity.

We investigated expression of TLR3 by western blot analysis in cell extracts from EBV-positive NPC tumor cells propagated *in vitro* (C666-1) or as xenografts (C15, C17, C18, C666-1) and in EBV-negative HONE1 and CNE1 cell lines comparatively to a panel of malignant and non malignant epithelial cell lines. Malignant cell lines were derived from HNSCC (SQ20B, FaDu), colon carcinoma (SW613), cervical carcinoma (HeLa, C33, CaSki), vulvar squamous carcinoma (A431), and non-small cell lung cancer (A549). NP69 and NP460 are immortalized non-tumorigenic cell lines derived from human nasopharyngeal epithelial cells. As shown in Figure 1A and 1B, TLR3 was consistently detected in NPC cell lines and xenografts, either EBV-positive (Figure 1A) or EBV-negative (Figure 1B). Its abundance was at a maximal level in extracts from NPC cells and xenografts followed by extracts from HNSCC cells (SQ20B and FaDu) ; in contrast, TLR3 was undetectable or at a low level in extracts from other carcinoma cells or from non-tumorigenic nasopharyngeal epithelial cells (Figure 1C). Modest levels of TLR3 were detected in CaSki and A431 malignant cell-lines, and in the non malignant nasopharyngeal cell-line NP460. On the other hand, we analyzed the *in situ* expression of TLR3 by immunohistochemistry in ten biopsies from primary NPC tumors. Positive and negative controls were C666-1 and NP69 cell pellets, respectively (Figure 2A). The TLR3 protein was consistently detected in NPC biopsies. In six out of ten specimens, most malignant cells were positive. In contrast, TLR3 staining was absent or weaker in stromal cells and tumor infiltrating lymphocytes. In the remaining four specimens, only a fraction of malignant cells scored positive, again with absence of staining or very low staining of infiltrating cells (Figure 2B, 2C and Table 1).

**Table 1:** Detection of the TLR3 protein by immunohistochemistry on tissue sections from NPC biopsies

**Patient N°**	**Origin**	**Gender/age**	**Clinical stage**	**TLR3 staining**	
				**Distribution**	**Intensity**
1	Tunisia	M/48	T4N3bM1	Homogeneous	Faint
2	Romania	F/34	T2bN0M0	Heterogeneous	Moderate
3	Cameroon	M/62	T4N2M0	Heterogeneous	Moderate
4	France	M/63	T4N0Mx	Homogeneous	Strong
5	France	M/62	T3N2M0	Heterogeneous	Strong
6	Portugal	F/84	T4N2M0	Heterogeneous	Strong
7	Algeria	M/40	T4N2M0	Homogeneous	Faint
8	Turkey	M/57	T1N0M0	Homogeneous	Moderate
9	China	F/31	T4N0M0	Homogeneous	Moderate
10	France	M/48	T1N2M0	Homogeneous	Faint

All specimens were of the non-keratinizing undifferentiated histologic subtype of the WHO classification (2005).

Images for cases 6 and 8 are displayed in Figure 2.

M0 indicates absence of extranodal metastases; M1, presence of extranodal metastases at the time of diagnosis; Mx, no assessment of distant metastases.

### The TLR3 agonist poly(A:U) and the Smac mimetic RMT5265 have synergistic cytotoxic effects on NPC cells

**Figure 3:**
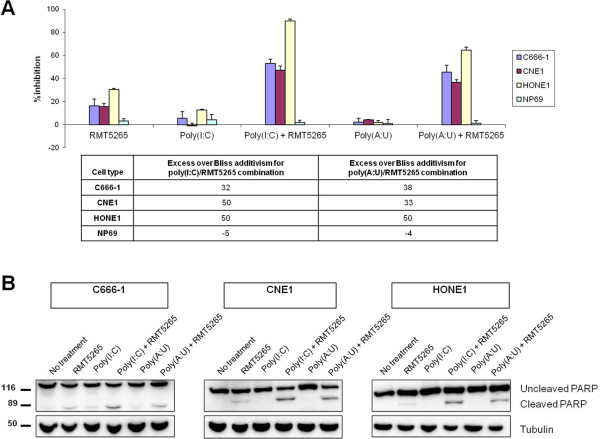
**Poly(A:U) and RMT5265 have cooperative cytotoxic effects on NPC cells.** (**A**) MTT cell-growth assay for NPC cells and non-tumorigenic nasopharyngeal epithelial cells treated with poly(I:C) or poly(A:U) (0.5 μg/mL for C666-1 and HONE1, 1 μg/mL for CNE1 and NP69) used either alone or in combination with a Smac-mimetic RMT5265 (5 nM for C666-1, 50 nM for CNE1, HONE1 and NP69). Poly(A:U) is known to have greater specificity for TLR3 than poly(I:C) which also stimulates the MDA5 and RIG-I receptors [19]. Despite this more restricted action, poly(A:U) combined with RMT5265 had cytotoxic effects on NPC cells almost equivalent to poly(I:C) and no effect on NP69 cells. Excess over Bliss additivism was calculated as indicated in the Materials and Methods section. The results are given as mean +/− SD. (**B**) Detection of PARP cleavage by Western blot analysis. Proteins were extracted after 24 h of culture from NPC cells treated with poly(I:C) or poly(A:U) used either alone or in combination with RMT5265. The drugs were used at the same concentrations as for the WST/MTT assays. 40 μg of protein extracts were loaded on PAGE gels. The blotted membranes stained with anti-PARP antibodies were also stained with anti-tubulin for protein loading control. No massive PARP cleavage was observed in any experimental condition. However the most abundant cleaved products were seen when poly(A:U) or poly(I:C) were combined with RMT5265

In a previous study, we reported the antiproliferative effects of a combination of the synthetic TLR3 agonist poly(I:C) and the Smac-mimetic RMT5265 on various types of malignant epithelial cells including NPC cells [36]. However, as already mentioned, poly(I:C) is known to stimulate not only TLR3, but also MDA5 and RIG-I, two cytoplasmic receptors of dsRNAs [19]. This is not the case for another TLR3 agonist, poly(A:U) [19]. Therefore, both TLR3 agonists were used, either alone or in combination with a Smac-mimetic (RMT5265), to assess their cytotoxic effects on NPC cells. Target cells were NPC cell lines (C666-1, HONE1 and CNE1) and non-malignant nasopharyngeal epithelial cells (NP69). They were treated for 72 hours with pharmaceutical agents used at low concentrations, ie less than 1 μg/mL for TLR3 agonists and 50 nM for RMT5265, and cell viability was subsequently assessed with WST/MTT assays. Poly(A:U) and poly(I:C) had similar cytotoxic effects on NPC cells when used in combination with the Smac-mimetic RMT5265 (Figure 3A). For example, in C666-1 cells, concentrations of RMT5265 as low as 5nM combined to poly(I:C) or poly(A:U) (0.5 μg/ml) were sufficient to achieve major cytotoxic effects. The combination of poly(A:U) with cisplatinum, namely the most widely used pharmacological agent in NPC treatment, revealed by contrast no more than an additive effect (data not shown). The TLR3 agonist/Smac-mimetic combination had no effect on NP69 non malignant nasopharyngeal epithelial cells (Figure 3A). To evaluate the contribution of apoptosis to the cytotoxic effects of the TLR3 agonists/Smac-mimetic combinations, PARP cleavage was assessed in treated cells. Malignant cells were exposed for 24 hours to poly(I:C)/poly(A:U) and/or RMT5265 at concentrations similar to those used for survival assays. No massive PARP cleavage was observed in any experimental condition, suggesting that the cytotoxic effects of treatment by single agents or combinations of two agents were only partially accounted for by apoptosis (Figure 3B). Nevertheless, a PARP cleavage of low intensity was detected in NPC cells treated with RMT5265 as a single agent. This cleavage was substantially enhanced when RMT5265 was combined to poly(A:U) or poly(I:C).

**Figure 4:**
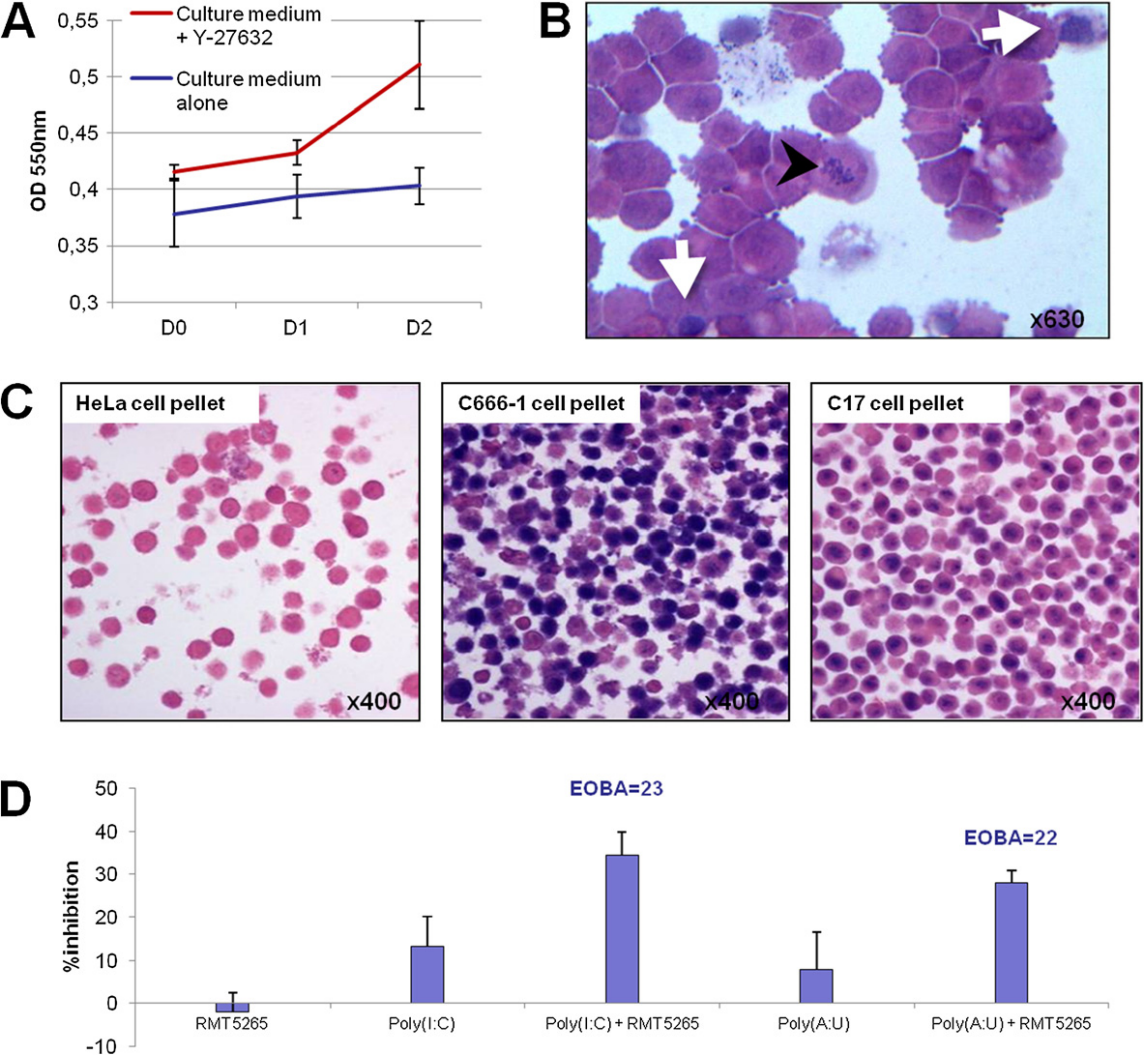
***In vitro *propagation of C17 cells and sensitivity to TLR3 agonists combined to RMT5265.** C17 NPC cells were dispersed from xenografted tumors and their propagation *in vitro* was made possible for the first time by adding a Rho kinase inhibitor (Y-27632) to the culture medium. (**A**) MTT assay was used to determine the growth characteristics of C17 cells cultured in media containing the Rho kinase inhibitor Y-27632 or not. (**B**) Morphological aspect of C17 cells propagated *in vitro* under optical microscopy. The aspect of some cells is evocative of mitosis (black arrowheads) and apoptosis (white arrows). C17 cells were stained with HES after cytospin preparation (passage 5 *in vitro*). (**C**) The positive EBV status of C17 cells was confirmed by EBERs hybridization, using HeLa and C666-1 cell pellets as negative and positive control, respectively. (**D**) MTT cell-growth assay on C17 cells treated with poly(I:C) or poly(A:U) (0.25 μg/mL) used either alone or in combination with the Smac-mimetic RMT5265 (200 nM). Excess over Bliss additivism (EOBA) confirms the synergic effects of these combinations. The results are given as mean +/− SD

Additional investigations were subsequently performed on C17 cells. These EBV-positive NPC cells which have been propagated as xenografts for many years were recently adapted to permanent *in vitro* propagation in our laboratory using a protocol described by Liu et al. (see Materials and Methods sections) [30]. This protocol which is proposed for primary cultures of a wide range of epithelial cell types, either malignant or non-malignant, has two main characteristic features: use of a feeder layer of non-transformed fibroblasts and addition of a Rho kinase inhibitor (Y-27632) to the culture medium. After the third *in vitro* passage of the C17 cells, we observed that the feeder layer was no longer required. In contrast, any attempt to withdraw the Rho kinase inhibitor resulted in a very rapid growth arrest (Figure 4A). The morphological aspect of C17 cells under optical microscopy is displayed in Figure 4B. The kinship of C17 cells propagated *in vitro* with C17 xenograft was confirmed by genotyping of HLA-A (HLA-A*02, *26) and HLA-B (HLA-B*44, *51) alleles. The EBV status of C17 cells propagated *in vitro* was assessed by EBERs hybridization on C17 cell pellets embedded in paraffin. HeLa and C666-1 cell pellets were used as negative and positive control, respectively. EBERs hybridization was positive, confirming that C17 cells retained the EBV genome when they were propagated *in vitro* (Figure 4C). Like all other NPC cells, C17 cells propagated in the presence of Y-27633 were exquisitely sensitive to the combination of TLR3 agonists with RMT5265 as shown by MTT assays (Figure 4D).

**Figure 5:**
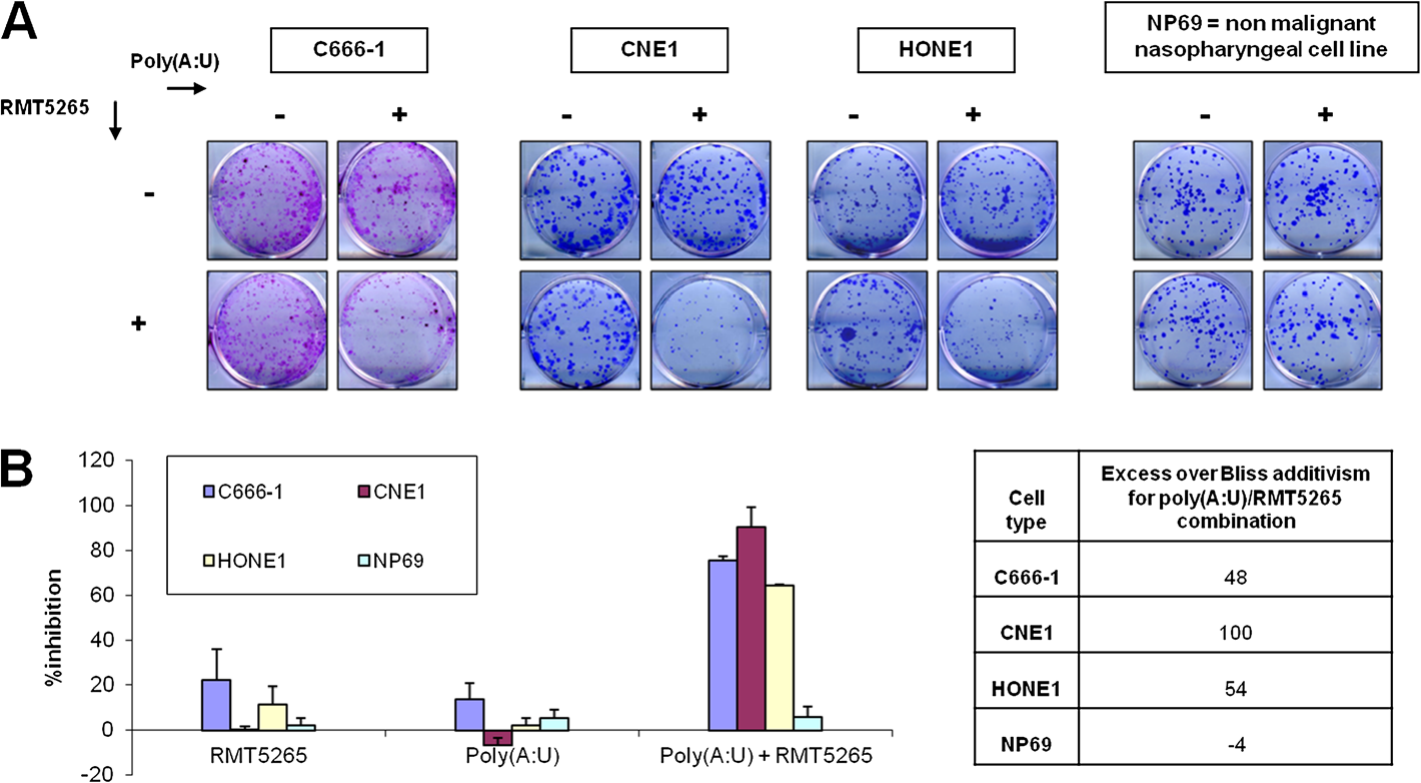
**Synergistic inhibition of NPC cells clonogenic growth by combination of poly(A:U) with a Smac-mimetic.** NPC cells and NP69 cells were treated with poly(A:U) (0.25 μg/mL for C666-1, 0.5 μg/mL for HONE1, 1 μg/mL for CNE1 and NP69) used either alone or in combination with RMT5265 (5 nM for C666-1 and HONE1, 30 nM for CNE1 and NP69). A feeder of irradiated Normal Human Dermal Fibroblasts (NHDF) was used for C666-1 clonogenic assays as described in the Materials and Methods section. (**A**) A representative aspect of the results of the clonogenic assays is displayed in the upper panel. (**B**) The percentage of inhibition was calculated based on the number of colonies, and the excess over Bliss additivism (EOBA) confirmed the synergic effects of the therapeutic combinations on NPC cells. The results are given as mean +/− SD

Finally, we assessed the effects of the poly(A:U)/RMT5265 combination on NPC cells clonogenic growth: concordantly with previous results, this therapeutic combination strongly inhibited the clonal growth of NPC cells, and had no effect on NP69 cells (Figure 5).

## Discussion

TLR3 overexpression has been reported in a number of human malignancies, such as melanoma, breast cancer, clear cell carcinoma, neuroblastoma, or head and neck squamous cell cancer [2,37-40]. To our knowledge, this is the first report of TLR3 expression in EBV-related NPC. The fact that TLR3 is strongly expressed by malignant NPC cells *in vitro* suggests that its expression is not a consequence of intra and peritumoral inflammation but is a constitutive feature of NPC cell phenotype. However, its expression is probably modulated by the tumor microenvironment. Investigations on tissue sections show that, in some fresh tumors *in situ*, TLR3 expression is homogeneous through all malignant cells whereas in some other tumors it is restricted to a subset of malignant NPC cells. Additional investigations will be required to determine whether TLR3-positive and TLR3-negative cells have distinct features in terms of proliferation and cell cycling, adaptation to hypoxia or influence of immune infiltrating cells.

The fact that TLR3 is consistently expressed by NPC cells suggests that it plays a role in NPC tumour growth. There are several potential natural endogenous ligands for TLR3 in NPC tumors. EBER RNAs are the first candidates. These viral non-coding RNAs are extremely abundant in NPC cells as in other types of latently EBV-infected cells; most of them are contained in the nucleus but a fraction of them can diffuse to the cytoplasm and the extra-cellular medium. In EBV-transformed B-lymphocytes, these extra-nuclear EBERs have been shown to bind TLR3 and to induce the production of inflammatory cytokines [41]. In NPC cells, the EBERs are known to induce the production of Insulin Growth Factor 1 (IGF1) which is itself an autocrine growth factor for NPC cells [42]. There is a suspicion that this effect is mediated, at least in part, by TLR3 [43]. That point will deserve further investigations. Beside the EBER RNAs, there are other potential ligands of TLR3 in tumor tissues. Messenger RNAs released by necrotic cells are known to bind and stimulate TLR3 probably as a consequence of the formation of secondary structures containing double strand domains [44,45]. The influence of necrotic messenger RNAs on NPC cell growth will also deserve investigations.

In an apparent paradox, several *in vitro* studies have underlined the potential of TLR3 as a therapeutic target for various types of human malignant cells [2,37,46,47]. However, the cytotoxic effects of TLR3 stimulation in the absence of another pharmacological agent, usually require extremely high concentrations of agonists, for example 10 to 100 μg/ml of poly(I:C). More recently, we and others have shown that it is possible to achieve a cytotoxic effect with much lower concentrations of poly(I:C) (in the range of 1 μg/ml) provided that the TLR3 agonist is combined with a Smac-mimetic [18,36,48]. Recently, the molecular rationale of this combination has been partially understood. Feoktistova et al. have described the “Ripoptosome”, an intracellular signaling platform containing caspase 8, caspase-10, RIPK1, FADD, and FLIP isoforms (short isoform FLIP-S or long isoform FLIP-L) that regulates cell death by apoptosis or programmed necrosis (necroptosis) in response to TLR3 ligands [48,49]. In this model, the c-IAPs prevent the formation and stabilization of the ripoptosome by ubiquitination and subsequent proteasomal degradation of a fraction of RIPK1 whereas FLIP-L inhibits the entry of RIPK1 and FADD into the ripoptosome following TLR3 stimulation. According to this model, the inhibition of the c-IAPs is believed to promote TLR3-induced cell death, either by caspase 8-mediated apoptosis or by RIPK1-RIPK3-mediated necroptosis, depending on the pre-existing levels of FLIP-S and FLIP-L.

In this study, we report that the combination of the poly(A:U) with the Smac-mimetic is as efficient as the combination involving poly(I:C). We also demonstrate that extremely low concentrations of both compounds - below 1 μg/mL for poly(A:U) and 50 nM for RMT5265 - are sufficient to achieve a significant cytotoxic effect. These data have important practical implications because poly(A:U) has distinctive characteristics which might be better suited for some patients than poly(I:C). It has a higher specificity for TLR3 and is believed to induce less systemic inflammatory effects [19]. It has already been evaluated in association with standard treatment in 5 phase III clinical trials [50-54]. Salaun et al. have reported that adjuvant treatment with poly(A:U) was associated with a significant decrease in the risk of metastatic relapse in TLR3 positive breast cancers [37]. In summary our data might have important implications not only for NPC treatment but also for other malignancies.

## Conclusion

We report that TLR3 is consistently expressed by malignant cells in NPC cell lines and clinical specimens. Low concentrations of the specific TLR3 agonist poly(A:U) combined to low concentrations of an IAP inhibitor have cytotoxic effects on NPC cells. These data confirm that TLR3 expression is a factor of vulnerability for NPC cells as it is the case for other head and neck carcinomas [40,55]. Additional investigations will be required to understand which type of growth advantage is associated with the consistent expression of TLR3 by NPC cells.

## Abbreviations

NPC: Nasopharyngeal carcinoma; HNSCC: Head and neck squamous cell carcinoma; TLR3: Toll-like receptor 3; RIG-I: Retinoic acid inducible gene I; PKR: Protein kinase R; Smac: Second mitochondria–derived activator of caspases; c-IAP: Cellular inhibitor of apoptosis Xprotein; XIAP: X-linked inhibitor of apoptosis protein; FLIP-L: Long isoform of the FLICE-like inhibitory protein.

## Competing interests

YM and CP are employed by Innate Pharma, a company developing a TLR3 agonist. All other authors certify that they have no conflict of interest.

## Authors’ contribution

BV and MG have performed most of the experiments. ASJP carried out NPC xenografts and shared her expertise in cell culture and Western blot analysis. YM and CP contributed the antihuman TLR3 mouse monoclonal antibody (40 F9.6, Innate Pharma, Marseille) for detection of TLR3 by immunohistochemistry. MW and PH have provided pathological samples for immunohistochemistry. MW, BV and PB have analyzed and interpreted stained tissue sections. KWL has provided the C666-1 cell line. SWT has provided the NP460 and NP69 cell lines. BV and PB designed the study, and drafted the manuscript. All authors read and approved the final manuscript.
